# Single-Port Laparascopic Cholecystectomy: Scarless Cholecystectomy

**DOI:** 10.1155/2012/204380

**Published:** 2012-05-07

**Authors:** Ahmad Mohmmad Zubaidi

**Affiliations:** General Surgery Division, Department of Surgery, King Khalid University Hospital and College of Medicine, King Saud University, P.O. Box 7805 (37), Riyadh 11472, Saudi Arabia

## Abstract

*Objective*. Single-incision laparoscopic surgery is a new advanced technology to further minimize the invasiveness of laparoscopy through a single small incision hidden in the umbilicus. The objective is to describe short and long-term outcomes of SILS cholecystectomy. *Methodology*. Patients with gallbladder pathologies were unselectively enrolled and were prospectively studied between April 2009 and April 2010 and completed a post-operative follow-up for 12 months. *Results*. There were 22 women and 8 men. Mean age was 46 years. Twenty-one patients had biliary colic, seven had acute cholecystitis, one had gallbladder polyp, and another had resolving acute pancreatitis. Mean operative time was 104.3 ± 44 minutes. Mean BMI was 30.42 and the average pain score was 3.2 ± 1.1. One more port was inserted to help in finishing the procedure in one patient. There was no conversion to a standard laparoscopic or open technique. There was one post-operative bile collection from a missed cyctic duct of Luschka. Mean hospital stay was 1.2 days. *Conclusion*. Single-port laparoscopic cholecystectomy is feasible. Early conversion to a standard laparoscopic technique is advised to avoid serious complications. The procedure has an obvious cosmetic benefit. Additional prospective trials are necessary to define the benefits of single-port laparoscopic cholecystectomy.

## 1. Introduction

In the journey of surgical access from a big incision to minimally invasive multiple keyhole ports, the road seems to be endless and full of innovative ideas and techniques. Nowadays, minimally invasive surgeons are solidifying their practice on transumbilical single-incision laparoscopic procedures (SILS) for what used to be done only through 4-5 access laparoscopic surgeries. There is a trend to perform operations without scars (natural orifice transluminal endoscopic surgery (NOTES)), [1, 2] a concept that encompasses a variety of techniques allowing the performance of complex operations without leaving visible evidence that surgery has occurred.

An editorial in *the Annals of Surgery* by Dr. Cameron and Gadacz, 1991, on the emerging popularity of the laparoscopic cholecystectomy attributed the rapid popular acceptance of the procedure as being “almost totally consumer driven” [3]. Twenty years later, conventional laparoscopic cholecystectomy has supplanted open cholecystectomy and became one of general surgery's “safest and most effective operative procedures”; however, emergence of the single-incision laparoscopic cholecystectomy as a new technique has won over “health care consumers.” Once again, surgeons are re-examining a gold standard in the face of a technological innovation. The first paper of SILS in 1997 described the use of two separate periumbilical incisions that were later connected for removal of the gallbladder [4]. Then in 2001, 70 laparoscopic cholecystectomies were performed with two trocars [5]. Currently, the literature describes the use of SILS techniques for multiple surgical procedures such as appendectomies, nephrectomies, adrenalectomies, splenectomies, colectomies, and varicocelectomies [1, 6–11]. SILS is a valuable addition to stealth surgery and seems to be ready for wider surgical applications.

This paper is the first to describe the long-term results of SILS for cholecystectomy on an unselected cohort of patients representing the reality of general surgery practice.

## 2. Materials and Methods

Thirty patients (22 women and 8 men) in this series were offered single-port laparoscopic cholecystectomy between April 2009 and April 2010. The average age of the patients was 46 years (range 24–96 years). Informed consent was obtained for the procedure from all patients, and the difference between the single-incision and the standard four-incision approaches was explained. All procedures were performed consecutively by the same laparoscopic surgeon with the assistance of a surgical resident. Study approval was obtained from the Institutional Review Board of King Khalid University Hospital. Data were collected prospectively for both quality assurance and subsequent analysis.

All patients had been evaluated for biliary disease either in the office or through the emergency room. Patients who demonstrated either symptomatic cholelithiasis, chronic biliary colic, biliary dyskinesia, or gallstone pancreatitis were enrolled, and surgery was scheduled on an elective or urgent basis, depending on the severity of the presenting disease. Patients with severe morbid obesity, who were pregnant, or whose American Society of Anesthesiologists (ASA) classification was 3 or 4 were not generally considered candidates for this approach.

Data analyzed included patient demographics (i.e., age, gender, body mass index (BMI), American Society of Anesthesiologists score), operative time, postoperative length of stay, and complications. Data presented are mean ± SD (range).

## 3. Operative Technique

**Figure 1:**
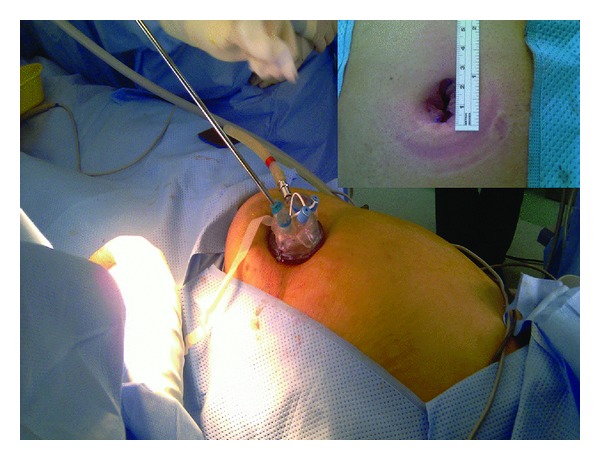
Operative placement of umbilical TriPort. In the small corners, the size of the skin incision does not exceed 2.5 cm.

All laparoscopic cholecystectomies were initiated as a single-site technique. The operation began with the injection of 0.5% bupivacaine into the umbilicus. For all the patients, access to the peritoneal cavity was made via a vertically oriented 20 mm incision through the center of the umbilicus using the direct Hasson technique; afterward, the single port was deployed into the abdomen [12]. The procedures were performed using a combination of straight and articulating instruments (Autonomy Laparo-Angle Instruments, Cambridge Endo, Cambridge Endoscopic Devices, Inc., Framingham, MA, USA) which allow six degrees of motion that correlated with the operator's wrist motion. An Olympus 5 mm EndoEYE video laparoscope was used for visualization (Olympus Europa GmbH, Wendenstrasse, Hamburg, Germany, Figure 1).

Following access and port placement, the operating surgeon and the assistant stood on the patient's left side. Our first exposure to the concept of single-incision laparoscopic surgery had come through the SILS Port (Covidien, Inc., Norwalk, CT, USA), and 15 of the patients for whom single-port laparoscopic surgery was attempted were offered the procedure using this device. TriPort (Advanced Surgical Concepts, Bray, Co, distributed by Olympus, Wicklow, Ireland) was used for the rest of the patients. A prior description of the mechanical aspects of these types of ports had been published [13]. The device is rotated so that there is a port at the 10, 5, and 2 o'clock positions [10]. One patient had previously undergone laparotomy. Adhesiolysis via the single port was successful enough to clear an operative field for safe visualization of the gallbladder and surrounding structures.

After the fundus of the gallbladder was visualized, a 2-0 Prolene suture on a straight needle was introduced through the abdominal wall using a technique described previously by Romanelli and colleagues [13, 14]. The suture was grasped and passed through the fundus of the gallbladder, then passed back through the abdominal wall. Traction on the suture, which was clamped at the skin level, retracted the gallbladder. This technique was used in one-third of patients in this cohort. No fundal traction suture was used in the rest 20 cases; the author found that procedure could be performed safely without it. Next, a reticulating grasper was used to retract the infundibulum to the right and slightly cephalad; then the handle of the grasper rotated to the surgeon's right side, away from the other instruments. The procedure usually began with a straight Maryland dissector or a hook with or without electrocauterization. The intention was to isolate the cystic duct and artery, clear the hepatocystic triangle, and separate the lower part of the gallbladder from the liver bed. This technique makes visible the cystic plate and enables the surgeon to have a critical, clear view, before clipping any ductal structures. Once the cystic duct and artery were clearly visible, both were double ligated with clips using an Ethicon Ligamax 5 mm clip applier and then transected with scissors. Electrocautery was used to remove the gallbladder from the liver bed, and the specimen was removed in a specimen bag along with the port. The fascial defect was then repaired with PDS sutures in a continuous fashion, and skin was closed with Dermabond (distributed by Ethicon, Inc., a Johnson & Johnson company).

## 4. Results

**Figure 2:**
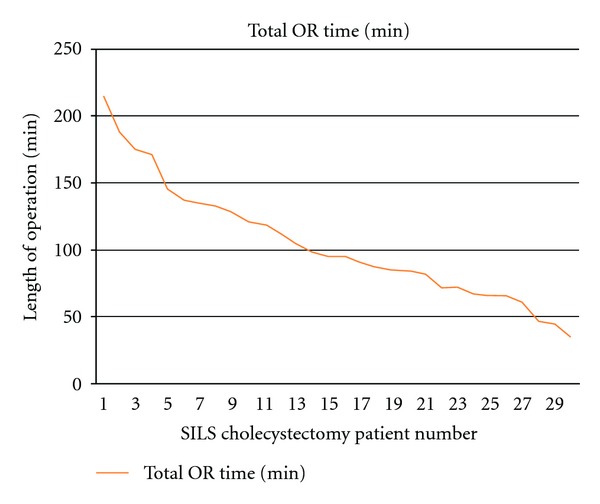
Length of SILS cholecystectomy.

None of the patients required an open operation. Twenty-nine patients underwent successful single-port laparoscopic cholecystectomy. We had to add another port to finish the procedure in a 60-year-old female patient with a resolving biliary pancreatitis because pancreatic head edema obscured safe visualization of the critical view of the Calot's triangle. There were more female than male patients in this study, as expected by the nature of the disease (22 women and 8 men; M : F 2.75 : 1) (Table 1). The mean age of the patients was 46 years (range, 24–96 years), and mean BMI was 30.6 kg/cm^2^ (range, 19.5–41 kg/cm^2^). The mean operative time, skin to skin, was 104 min (Figure 2). The estimated blood loss in all patients was ≤50 g. There were no intraoperative complications. Average pain score was 3.2 ± 1.1 postoperatively. The length of the post-operative hospital stay was 2 ± 1 days. There was no wound infection, and no mortality was observed.

**Figure 3:**
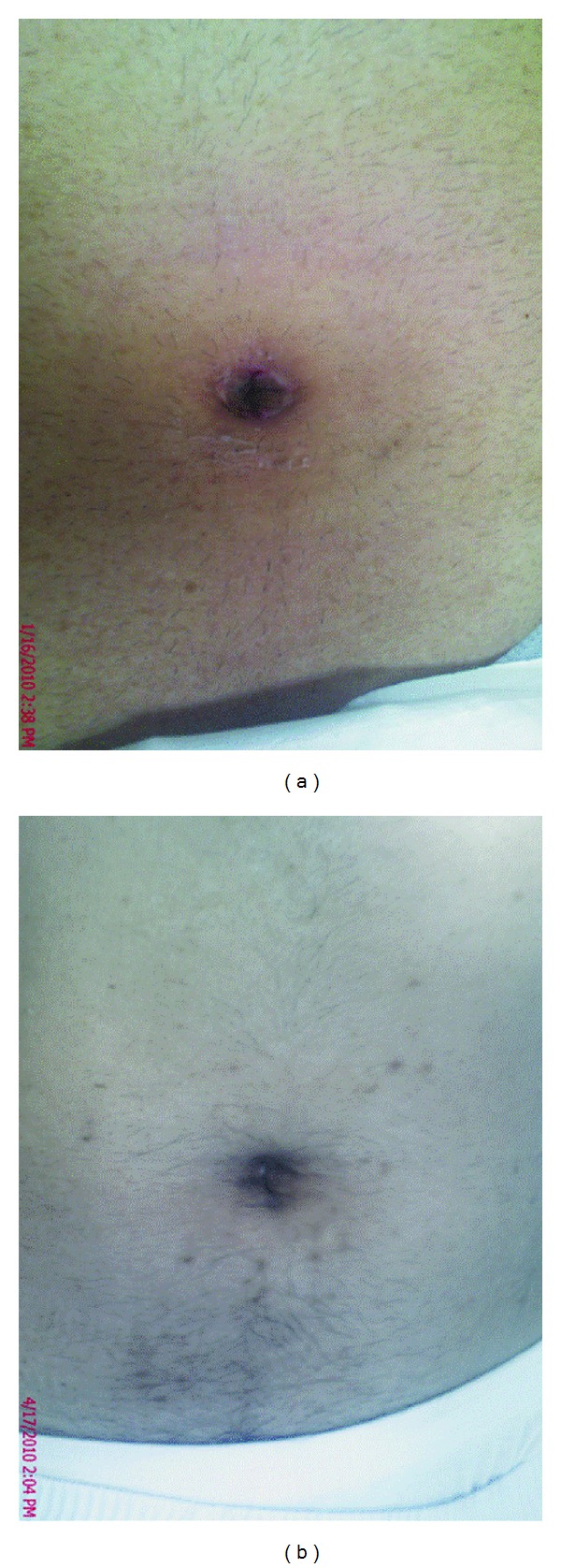
Views of umbilical scar after surgery.

One patient had to be readmitted for continuous postoperative abdominal pain which was found to be attributable to a bile collection at the gall bladder fossa. The patient underwent ERCP which delineated a missed accessory bile duct in the gall bladder fossa (duct of Luschka). The problem was managed with a temporary stenting of the common bile duct. No injuries to the main biliary tree were recorded in this series. All umbilical incisions were concealed within the umbilicus. There were no records of umbilical wound drainage or infection in the short-term followup. All patients were followed for 12 months. There was no umbilical hernia recorded. Cosmetic outcomes at followup were excellent with a minimal, barely visible scar in most patients (Figure 3).

## 5. Discussion

In the recent years, laparoscopic surgery has developed rapidly. Although Navarra and colleagues [4] reported SILC 14 years ago, the procedure did not gain wide acceptance until a decade later because of great technical progress and remarkable improvements in the handling of the instruments and visualization. Single-incision laparoscopic surgeries are increasingly described as potentially less invasive, “stealth” procedures and have recently been performed for many intra-abdominal pathologies such as appendectomy [15], adrenalectomy [16], gastric banding [17], and donor nephrectomy [18].

Cholecystectomy is a procedure with a low morbidity rate worldwide. An important factor with laparoscopic approaches to the gallbladder is the ability for the surgeon to obtain a “critical view of the Calot's triangle.” Most surgeons who routinely perform laparoscopic cholecystectomy would consider the critical view as a basic requirement and would be greatly concerned by any new technique that compromised it. Departure from some of the basic tenets of laparoscopic surgery is a major disadvantage of this operation. Most important is the virtue of placing both the laparoscopic camera port and all dissecting instruments through a single umbilical incision, causing lost of triangulation between the camera and the working ports [19]. This leads to collision of instruments, cross-handedness, and restriction of movement and viewing, as well as dissecting angles. In addition, placing a suture directly through the gallbladder to provide retraction and exposure leads to some degree of bile spillage from the suture punctures with this technique. Because of instrument collision and cross-handedness, we tended to struggle at the beginning of our experience. The surgeon must cross hands to obtain a reasonable angle of distraction of the tissues in the operative field. However, in all cases in this cohort of patients, the critical view required was obtained, using a combination of traction sutures, an articulating grasper, and bendable angled laparoscope. When the critical view was compromised in one of our patients, an additional port was added to help in visualization of this view. Thus, the critical-view principle was followed.

This study was performed nonselectively on all presentations of biliary disease, whether acute or chronic. We shared some of the contraindications considered by Kuon et al. [20] such as a BMI >30 kg/m^2^, suspicion of a malignancy, and the presence of a cystic duct stone. However, acute cholecystitis and previous upper abdominal surgery were not considered contraindications to our group.

Our mean operative time was 104 minutes, longer than the time required for classical 4 ports cholecystectomy. The extra time reflected the degree of the procedure complexity and the learning curve of the operating surgeon, and there was a trend to decreasing operative time as more cases were done.

All the patients had normal liver function tests, a normal common bile duct diameter on ultrasound imaging, and no anatomic questions at the time of surgery. Therefore, cholangiography was not indicated in this series and was not considered. We share the same concern as other authors on whether the approach from the umbilicus would be appropriate for cholangiography and how clear the ultimate image obtained would be, although successful use of cholangiography with a single-port approach has been reported previously [21]. Adding cholangiography would certainly increase the operative time.

We did not observe any increase in pain levels or more consumption of analgesic medication either during admission or in the two-week outpatient followup in this series. This observation is in concordance with a previously published report [22].

Preincisional wound infiltration with a local anesthetic seems to have provided some benefit in early postoperative pain reduction [23, 24].

There was one readmission in this study, a bile collection from an accessory bile duct leak (duct of Luschka), which was managed conservatively with CT-guided drainage of the collection and a temporary endoscopic decompression of the common bile duct. No patients in the series experienced complications related specifically to the cholecystectomy (i.e., cystic duct stump bile leaks, ductal injuries, bowel or liver injuries).

All patients completed an outpatient followup for 12 months postoperatively. Our protocol was to see them in the first two postoperative weeks and then every three months until the end of the 12th postoperative month. The procedure of single-port cholecystectomy left a barely visible scar in most patients. It provides the same benefit of scarless surgery of NOTE as the incision is well hidden in the umbilical cicatrix, which in itself is an embryological natural orifice (Figure 3).

An incarcerated hernia at the site of the single incision has been reported in another study [19]. This is an alarming complication. It suggests that incarceration certainly is more possible with a larger fascial opening. However, this incision is not larger than the incision for a standard 12 mm trocar site and should be compared with it. For this reason in specific, we closed the fascial with # 0-PDS suture in a continuous fashion with no fascial strangulation and elected to follow up our patients for 12 months to observe the incidence of incisional hernia. Fortunately, no incisional hernia was observed by our group or has been documented in our patients by other physicians.

In conclusion, we submit that single-port cholecystectomy is feasible, safe, and possible in most cases of cholelithiasis. A fundal stitch for retraction may and should be used whenever visualization of the Calot's triangle is suboptimal. Single-port cholecystectomy has an obvious cosmetic benefit over standard laparoscopic cholecystectomy. It may offer an acceptable alternative to NOTES. However, additional prospective trials are necessary to define these benefits and to determine whether this can be recommended as a standard procedure.

## Figures and Tables

**Table 1:** Summary of patient characteristics.

Patient characteristics	Conventional SILS (*n* = 30)
M : F	2.75 : 1
Age (years)	46 years (24–96)
BMI (kg/m^2^)	30.596 kg/m^2^ (19.5–41)
Operative time (min)	104 min (35–215)
Blood loss (g)	20 ± 15 mL
Postoperative hospital stay (days)	2 ± 1 days
Wound complications	Nil
Other complications	Biliary leak from duct of Luschka

M: male, F: female, SILS: single-incision laparoscopic surgery, BMI: body mass index.
